# Learning from adaptation to develop loss and damage monitoring and evaluation systems

**DOI:** 10.1038/s44168-026-00384-0

**Published:** 2026-05-15

**Authors:** Susannah Fisher, Lisa Vanhala

**Affiliations:** 1 Department of Risk and Disaster Reduction, University College London, London, UK; 2 Department of Political Science, University College London, London, UK

**Keywords:** Climate sciences, Environmental studies

## Abstract

Loss and damage is now firmly entrenched in global climate governance, yet there has been relatively little discussion around how to determine whether a loss and damage intervention is successful. We review the conceptual, methodological and political challenges identified within adaptation monitoring, evaluation, reporting and learning (MERL) to draw lessons for responding to loss and damage. Our review concludes by laying out a research and action agenda.

## Introduction

Monitoring, evaluation, reporting and learning systems (MERL) for adaptation have received considerable research attention over the past decade^1–6^, as part of a move to results-based management reflected also in international frameworks for disaster risk reduction, biodiversity and sustainable development^7,8^. In recent years, loss and damage has emerged as a new policy agenda alongside mitigation and adaptation in the United Nations Framework Convention on Climate Change (UNFCCC). Loss and damage (L&D) as a term refers to the negative consequences that arise from unavoidable risks of climate change that are beyond adaptation limits^9^. The emergence of L&D as a policy and scientific agenda is based on the recognition that adaptation alone is no longer sufficient to protect those most vulnerable to climate change and that as limits to adaptation are now being reached, losses and damages are being experienced by communities around the world. Loss and damage is often framed as part of a justice-based agenda, and some argue justice should be an integral part of responding to losses and damages and assessing these responses through MERL^10,11^. As the institutional frameworks are put in place to channel finance for loss and damage through the Fund for Responding to Loss and Damage and other bilateral and multilateral sources, the research and practice community around loss and damage have begun to face many of the challenges the adaptation community have been addressing over the past two decades (e.g., ref. ^12^).

In this review, we first outline the context for MERL systems in adaptation and loss and damage. We then draw lessons from scholarly literature and practice on adaptation MERL to analyse the conceptual, methodological and political challenges that researchers, practitioners and policymakers should be aware of when developing MERL systems for loss and damage. We go on to analyse the cross-cutting lessons and develop a research and action agenda that proposes areas for future research, theoretical development and institutional reflexivity.

### Context of MERL systems

Scientific attention on adaptation MERL has been consolidated through conclusions of the Sixth Assessment Report (AR6) of the Intergovernmental Panel on Climate Change (IPCC) that emphasised the critical importance of such systems and the additional focus on effectiveness as well as through the planned update on the 1994 Technical Guidelines for Assessing Climate Change Impact and Adaptations in the AR7 cycle. In parallel, there have been international policy developments that have catalysed attention on monitoring, evaluation and learning processes, such as the global goal on adaptation and the global stocktake in the UN processes. This research and the related policy developments build on almost twenty years of practical experience developing MERL systems for adaptation by a range of actors including NGOs, governments, and multilateral and bilateral agencies. The literature shows a trajectory that develops from initial conceptual papers^13,14^, to discussion of the emerging challenges^1,4^, proposed new methods and frameworks^15^(see a review^16^) and more recently a critical lens on what these forms of measurement have achieved and how they might shape adaptation action^17–20^.

New approaches to loss and damage are being designed by policymakers and practitioners and the question of how to monitor, evaluate, report and learn from these initiatives is growing in importance. Practitioners are considering alternatives to traditional project-based approaches^21^ and can benefit from early lessons from emerging institutional responses to addressing loss and damage. In addition, some of the early loss and damage projects are beginning to see outcomes^22,23^. For example, the Scottish Government was the first to explicitly commit funding to addressing loss and damage. Between 2022 and 2023, it funded projects, inter alia, to support research projects focussed on operationalising loss and damage finance and developing case studies of efforts to address loss and damage in Malawi, Rwanda and Zambia^24^. As these results emerge, understanding how to evaluate whether these projects are successful in ways that are proportionate, and reflect the long-term continuous nature of the outcomes is crucial^22^. The role a MERL system can play may also shift over time as systems mature and move from simply tracking outputs or assessing single projects, to a learning system anchored within institutions. Our focus in this review is the cross-cutting challenges and questions that MERL processes present, which are found within specific projects but also national frameworks and global assessments. Our intent is the analysis will allow policymakers and practitioners to consider what scale of assessment is appropriate.

The relationship between adaptation and socio-economic development is a complex one, making it challenging to manage the boundaries between the interventions to address them in many locations^18,25^. The contextual and diverse nature of adaptation also means universal definitions and approaches can rest on significant assumptions that don’t apply equally in all contexts^26^. We argue both these challenges will also apply to loss and damage. The variable nature of the data and evidence available on economic and non-economic losses, as well as climate data and socio-economic data systems, risks entrenching epistemic inequalities across both agendas^27^. Despite all of these challenges, tracking and evaluating progress has the potential to play a role in reducing vulnerability to climate impacts through informing both adaptation and loss and damage planning, funding and implementation priorities^28^. Learning and institutional reflexivity have been identified as key dimensions of effective MERL processes for adaptation although current systems often struggle to focus effectively on these dimensions^29–31^.

Scholars have usefully considered how lessons from allocation of adaptation finance can inform loss and damage decisions^32^. In this review, we take this further, seeking to understand how lessons from research on adaptation monitoring and evaluation systems can inform how loss and damage researchers and practitioners can measure the effects of interventions put in place to respond to loss and damage. Challenges to MERL systems in adaptation have been identified in three main domains: conceptual, methodological and political^1,28,33^. In the following sections, we structure our review around these three categories. First, we analyse the relevant conceptual debates on adaptation MERL systems and loss and damage to draw out areas where loss and damage scholars can learn from evidence from adaptation and identify areas where it presents genuinely new conceptual challenges. Second, we turn to the literatures on methodological challenges and highlight some of the specific issues that are likely to arise in the case of loss and damage. Third, we discuss the political challenges, highlighting some of the overlapping issues that are emerging between adaptation and loss and damage MERL.

### Conceptual challenges

#### Defining a policy area

Early debates on adaptation focussed on the relationship between adaptation as an additional activity to address climate risk and as a socio-economic development, a process that should decrease poverty and so vulnerability to climate risks^25^. Spearman and McGray^14^ developed a spectrum of adaptation action ranging from addressing the development deficit, to reducing vulnerability, managing current climate variability and then finally addressing the additional and future risks from climate change. Making this distinction between adaptation and development was particularly important in the context of the international climate finance regime where funding was agreed to be additional to overseas development assistance^34^. This led to much early debate on how to conceptualise outcomes from adaptation initiatives in a way that would be separate from development outcomes such as poverty reduction (see ref. ^35^).

The spectrum of activities that respond to risk were again extended with the development of the concept of loss and damage beyond the limits to adaptation. Limits to adaptation are the point at which an actor’s objectives (or system needs) can no longer be achieved through adaptation action due to the continuing residual risks from climate impacts^9,36^. Hard adaptation limits are cases when intolerable risks cannot be avoided through adaptation efforts. Soft adaptation limits refer to instances where options for adaptation may be able to avoid intolerable risks, but they are currently not available. This can be due to financial, institutional, or governance reasons amongst others^37^. Some scholars are now seeking to further understanding of these concepts, recognising their importance in the coming decades^38^, and research with local actors shows “that adaptation barriers and limits are not the same for everyone”^39^ (p9).

The conceptual challenges around loss and damage are considerable and relate to the relationship to adaptation and other related domains such as disaster risk management, humanitarian action and biodiversity loss, to name a few. Theoretical developments in loss and damage research and developing comprehensive action to address it in practice have been made even more challenging by this lack of conceptual clarity^40–42^. The IPCC distinguishes between two terms used in these debates: Loss and Damage (title case), referring to political debate under the UNFCCC, and losses and damages (sentence case), referring broadly to harm from (observed) impacts and (projected) risks, both economic and noneconomic^43^. There is no agreed UNFCCC definition. In practice, the adverse effects of climate change that are not or cannot be avoided by mitigation and adaptation efforts is how loss and damage is most commonly understood^44^. What is becoming clearer as practitioners engage with loss and damage as a policy domain is that many loss and damage interventions will address needs which occur beyond the limits of adaptation: the material and intangible consequences of climate impacts.

Understanding and defining the relationship between adaptation, adaptation limits, maladaptation and loss and damage will be important for designing L&D MERL systems as well as for evolving adaptation MERL in the context of L&D. The lack of clarity in the definition of both adaptation and loss and damage will have other implications as measurement systems needs to assess progress whilst also maintaining a distinct policy area. This has implications for what it is understood to be useful to measure, and what outcomes come to be seen as most important. There are risks to missing the connections and inter-linkages between policy areas.

#### Measuring changes in outcomes

How to conceptualise the outcomes of adaptation interventions has been an area of active scholarly debate and practical discussion amongst funders and those implementing adaptation projects. Conceptualising changes through the tracking of policy progress or institutional readiness to adapt has been one approach^17,45^. For example, some institutional indicators are reflected in the framework for the global goal on adaptation and provide a planning trajectory for governments and other actors to follow through the adaptation policy cycle. A variety of concepts and frameworks have been developed to assess outcomes for households, communities and systems drawing on the early IPCC framing of vulnerability as a function of adaptive capacity, sensitivity and exposure^46^. These proved challenging to measure and operationalise in different contexts^5,32,47^. Some practitioners argued for outcome measures that focused on achieving development objectives despite the additional climate risks, with measures such as resilience, adaptive capacity and decreasing vulnerability only seen as intermediary steps to that goal^48^. However, the practicalities of multilateral and bilaterally funded projects, where attribution of a project output to an intended outcome has been an important principle, combined with the desire to keep adaptation and development conceptually distinct for funding and accounting purposes has shaped measurement choices. Many measures deployed by bilateral and multilateral funds and agencies have focused on, for example, counting beneficiaries reached (variously defined), land or assets sustainably managed or rehabilitated, and the institutional processes needed to be in place for adaptation to occur (e.g., capacity, plans, awareness)^49–51^. Despite these measures for reporting, there has been widespread agreement that very little is understood about the long-term effectiveness of most adaptation interventions^52–54^. The debate over how far to use universal metrics for adaptation that allow aggregation and comparison but require a set of assumptions about local contexts and outcomes for adaptation has been ongoing^26^.

Resilience has become a core concept in measuring outcomes of adaptation projects and has been widely used by bilateral and multilateral agencies to assess change of individuals or households over time. One widely used metric in adaptation projects has been the number of people anticipated or assumed to become more resilient, often operationalised as the number of people reached by the project^54^. This has led to large numbers being reported, but the conceptual coherence within the reported numbers is often very fragmented giving little insight into any actual changes in people’s lives. Debates have focused on how to measure resilience^55,56^ as well as wider geographical work that has critiqued the neoliberal nature of the concept and the individualisation of risk^57,58^.

Like adaptation, the outcomes of responses to loss and damage can be conceptualised in different ways and this will shape how success is understood. The most commonly used distinction in defining loss and damage has been between economic loss and damage (e.g., income, physical assets, economic natural assets) and non-economic loss and damage (e.g., societal, individual and environmental). Economic loss is more immediately amenable to existing metrics and quantified datasets. Recent literature has offered new innovations in thinking about how to understand loss and damage. There is a growing emphasis on lived experiences and centring contextual values (see refs. ^27,59,60^). Recent work has also challenged the dichotomy between economic and non-economic loss and damage noting that research aimed at informing policy must be better attuned to the multifaceted and cascading nature of loss and damage^60^. Emerging literature on vulnerability dynamics (e.g., ref. ^61^) provides some ways to consider the cumulative nature of how households and groups experience climate shocks and stressors.

These complex understandings of what loss and damage mean to different communities echo similar debates on the contextual specificities of measuring resilience in adaptation. The adaptation field has moved on pragmatically, using aggregated portfolio assessments within bilateral and multilateral programmes for international audiences and capturing local contexts through individual project log frames and local or regional learning agendas. This raises questions for scholars and practitioners working on loss and damage as to how to balance the competing calls for locally specific knowledge and value-driven approaches with the political pressures that often result in top-down results frameworks^62,63^, and where to focus their attention and effort.

As well as the similarities with adaptation, the question of outcomes for loss and damage responses has some conceptually distinct elements – one of these is the use of time. Within adaptation, measures seek to create capacity or resilience to respond to future events. In loss and damage, many interventions are responding to risks that have already materialised. This means scholars and practitioners will need to consider the timeframe a response is designed and sustained for and whether it also seeks to be future-oriented to new and cascading risks.

### Methodological challenges

Beyond the conceptual challenges, adaptation presents certain methodological challenges to MERL due to the long timeframes over which interventions need to have impact, the uncertain nature of future climate impacts, and the changing baselines from shifting hazards^15^. Many of these challenges will also apply to L&D such as the changing nature of future loss and damage and the long timeframes over which the impacts of any response to loss and damage will be felt, as we outline in Table 1. Measuring long-term outcomes and the sustainability of any outcomes have been a key issue in adaptation planning and, therefore, in MERL. This had led to a diversity of approaches, with some indicators focusing on measuring the enabling institutional environment and some focussing on the anticipated outcomes of a project^28^.

**Table 1:** Key questions for MERL of responding to loss and damage

Challenges	Approach taken in adaptation	Emerging questions for responding to Loss and Damage
Conceptual	• Defined as separate from socio-economic development to support separate funding streams. • Institutional tracking of milestones • Complex resilience and adaptive capacity concepts and number of beneficiary metrics not created robust evidence base and prioritised aggregation	• What is gained and what is lost by defining loss and damage as an entirely separate category from adaptation? • How will the boundary be measured and might projects move back and forth over the threshold over time? • How can institutional tracking go beyond symbolic performance? • Will it be important to aggregate results on responding to loss and damage or will local understandings be prioritised? • How can different types of loss be understood?
Methodological	• Shifting baselines and hazards • Long timeframes for impact • Lack of data and alignment with national systems	• How do outcomes compare to those left in potential worsening contexts? • What does sustainability of outcomes mean in the context of responding to loss and damage? • How will loss and damage data systems link with adaptation?
Political	• Defining success of an intervention • Incentives to report success	• What incentives does any system create? • What does success look like to different groups?

Researchers have emphasised the importance of being specific about the pathways to change for adaptation - showing how any intervention needs to be explicit about what hazards it responds to, over what time period^48^. Attributing a specific outcome to one investment has proven challenging due to the complex environments in which most adaptation occurs with many factors shaping outcomes for households and communities. Some MERL systems, therefore, focus on contribution instead. The issue of shifting hazards and baselines has been addressed in some projects through asking implementers to give climate narratives alongside their results or looking at outcomes before and after specific events^64^. This has been data intensive and required high levels of capacity that was not highly scalable. The UN Adaptation Fund has used ex-post evaluations as a way of understanding the longer-term effects of interventions and scholars have proposed methodologies to support ex-post assessments^65,66^. There is a move within adaptation MERL to propose approaches based on systems innovation and citizen-generated data, although there has been limited implementation of these to date^67^.

As well as MERL systems for specific projects or programmes, they can also be developed for national policies and global assessments of progress. At these scales issues of coordination and existing data availability become increasingly important. International programmes such as the Climate Investment Fund’s Pilot Programme on Climate Resilience (PPCR) have provided specific support for national governments to set up systems to assess their progress towards adaptation goals. Such systems are often designed as part of wider policy processes that include assessment of key data on vulnerabilities and hazards. The monitoring frameworks often include assessment of institutional milestones such as policies, awareness and coordination mechanisms. Frameworks such as the locally-led adaptation principles offer another way of assessing progress towards the enabling environment^68^. In some cases, there is limited or fragmented national data available for national assessments and countries have been unable to operationalise adaptation MERL systems due to their complexity^69,70^. When countries do have indicators in their national plans, they often do not track them or do so only symbolically due to these challenges^17,45^.

There has so far been little specific debate on the practicalities of loss and damage measurement systems (but see ref. ^12^ for an exception), although by our analysis they will throw up many of the same methodological issues as in adaptation. Those developing loss and damage MERL systems will need to consider what kinds of frameworks, data and evidence can be used to assess (1) when the limits to adaptation are being reached and result in loss and damage, and (2) whether responses to loss and damage are effective, efficient and seen as legitimate by relevant stakeholders. This gets at the important distinction between using MERL to assess loss and damage versus using MERL to assess responses to loss and damage. In the case of the former, the initial methodological challenge will be establishing that threshold as the limit in many cases could be soft, and also value-driven. For example, one individual might feel their home is uninhabitable due to flooding, whereas another individual may not reach that same conclusion. For post-impact loss and damage response evaluations, the baseline of a loss and damage intervention could be the wellbeing or the perceived wellbeing of a community. This will also change over time and may be less sensitive to shifting climate hazards as by definition it will be responding to a threshold that has already been crossed. However, following recent work on measuring wellbeing after planned relocation (see ref. ^71^), these changes in community outcomes need to be considered in the context of what outcomes would have been if communities stayed in an increasingly risky location rather than their situation immediately before (or after) a relocation. The issues of the non-finite nature of loss^72^ and sustainability are also likely to be important, and here the loss and damage community can learn from the challenges in instigating long-term change through adaptation investments, and how those long-term impacts can be measured through ex-post assessments.

Any MERL system will need to be attentive to the assumptions of how change happens associated with a project or programme and new theories of change will need to be developed for some types of loss and damage^73^. They will vary across scales of governance, from the local to the national and global, as well as between different ways of averting, minimising or addressing loss and damage. Data gaps are considerable within adaptation, and loss and damage will face similar challenges. Any system for loss and damage needs to balance the incentive to measure impact with how and to whom the potential benefits to this knowledge will be accrued.

### Political challenges

A final area where research has identified challenges within adaptation MERL systems is the politics of defining what success looks like, and the incentives metrics and frameworks create. The UNFCCC global stocktake asks countries to assess whether adaptation is adequate and effective implying a single, objective definition of effectiveness^6,74^. However, research on adaptation success shows it is a political concept^33^ and visions of success are linked to how stakeholders view the future^75^. The complexities of the politics of measurement have been demonstrated recently with the international process to develop the framework and then indicators for the global goal on adaptation^76^. 59 indicators (from a proposed list of 100) were adopted at COP 30 in 2025 that cover both institutional risk management and results in outcome areas such as health, infrastructure and cultural heritage. Research by Singh and colleagues^18^ drew out eleven approaches to adaptation that shaped what effective adaptation looked like to stakeholders. As well as the politics of defining success, there is also a wider politics to measuring success of an intervention funded through external sources where continued success is perceived to be a necessary requisite for further funding at the national level. In this context, openly demonstrating how parts of the project have not worked is perceived to undermine chances of future finance and so MERL systems risk creating incentives to demonstrate progress rather than report any failures. The indicators also create incentives and norms, which partly define the policy issue at hand and what might be suitable solutions^19,62^. Whilst the results-based paradigm is currently dominant within public policy (see refs. ^7,77^), other scholars show how this is a relatively recent approach and past UK governments, for example, invested overseas development funds towards broad multilateral objectives rather than requiring a specific results-based framework^78^.

Loss and damage has been a highly politicised agenda as it has emerged within the inter-governmental negotiations of the UNFCCC^42^. Although loss and damage were explicitly established in the Paris Agreement there has so far been little debate on what a successful loss and damage intervention might look like, and what constitutes ‘success’. The boundaries between some adaptation measures and loss and damage responses continue to be blurry. But for those loss and damage responses that are designed to support communities once a threshold or limit has been reached, there is no obvious test for adequacy or way to assess when loss has been sufficiently recognised. A related area of debates on loss and damage responses – policies, projects and programmes including metrics – has argued they can offer opportunities for transformational change^79^. For example, Morrissey and Oliver-Smith^80^ argue it will be important to capture “the opportunity for transformation in the way loss and damage is assessed and addressed” (p. 3). Scholars have argued that people and their lived experiences of loss and damage need to be at the heart of the analysis, leading to a situated and socially engaged science of loss^81,82^. As Tschakert and colleagues^27^ note, this is part of “an attempt to counter the material and discursive erasures evidenced in quantified top-down, expert-driven, and context-detached assessments” (p59).

The politics of adaptation and loss and damage create challenges in generating transparency around what works and what doesn’t, with incentives aligning to make the policy area measurable through traditional results-based management approaches and demonstrating successful implementation rather than try new riskier approaches^53^. The emerging science of loss points to a different approach to MERL and this will require a concerted effort to support and a shape a new politics of results-based management to that within the international development and adaptation community. Many of the debates on loss and damage around locally specific understandings and value-driven approaches echo debates on adaptation. However, the MERL frameworks that have been widely implemented in adaptation have often had to comply with external needs for information, accounting and aggregation, and have not created substantial evidence on issues such as longer-term outcomes (with some notable exceptions). The loss and damage community will need to decide on their priorities and advocate for them within the MERL structures of loss and damage funds, projects and programmes if they wish for a different outcome.

### Learning from adaptation for loss and damage

When designing a loss and damage MERL system, it is important to consider the purpose and the audience. For example, the purpose of a system might be learning, reporting, evaluative findings or a personally transformative experience for those involved. Each of these purposes will lead to trade-offs between the complexity of the data that is collected and the wider use of that information by different stakeholders. Whilst we explore many lessons from adaptation MERL and the emerging science of loss above, we also stress the need to be pragmatic and cautious of creating parallel reporting systems that are too complex to be implemented, resulting in symbolic performance^45^. L&D MERL systems will need time to develop in maturity. Initial systems will need to focus on creating the enabling environment for MERL - this might include engaging stakeholders, developing and testing indicators or frameworks including measures of loss, building baselines, setting up monitoring, and identifying the use case for the data. Institutional tracking or assessments of policy progress can be useful proxies for change in early frameworks but have their limitations. Over time, systems might focus on feeding back into project design, assessing longer-term outcomes and evaluative data, as well as integration into wider M&E and socio-economic systems. Transformative learning for managing and addressing loss and damage, as scholars have been arguing for, might be developed in these later stages.

Building on the review, we summarise in the table below the key questions that the adaptation research on MERL has opened up for the loss and damage community. We argue that this moment of inflection in loss and damage programming is a vital time for scholars, practitioners and policy makers to consider what has worked and what hasn’t in the 20 years of experience of adaptation MERL to ensure the results frameworks designed for L&D reflect the emerging science of loss and damage.

Some areas of loss and damage present different challenges to MERL than those posed by adaptation. These include domains such as post-loss support, commemoration, and recognition of loss. Here, scholarship on transitional justice that offers concepts such as restitution, satisfaction (symbolic measures) and guarantees of non-repetition may offer potential ways ahead^83^ as well as drawing on practical experience and grey literature from sectors including cultural heritage, psychological support and post-trauma rehabilitation.

### Towards a new research and action agenda

We conclude the review with three emerging areas on loss and damage MERL that merit further research and analysis building on our analysis in Table 1. These areas reflect future research needs, areas of theoretical development and areas where institutional reflexivity will be needed to support more effective and equitable approaches to loss and damage.

Firstly, understanding how the threshold between adaptation and loss and damage will be measured and maintained – and the effect of this boundary work on implementation – will be an important area for academic enquiry and learning through practice. The threshold where adaptation moves to loss and damage, from both a practical and scientific perspective, will need to be carefully considered as part the design of a MERL system. One location or community may have a loss and damage project following an adaptation one or overlapping with one. Some adaptation projects are already documenting loss and damage in their MERL systems and this can be an important source of evidence for new L&D projects and MERL systems. There are emerging debates on related issues such as adaptation limits that will be important for loss and damage scholars to engage with as part of this work (see refs. ^38,39^). If governments choose to develop national policy and MERL frameworks separately for loss and damage and adaptation there will need to be attention to how the two areas relate to each other, and to the effects on both agendas of maintaining a firm boundary with the related but separate epistemic communities, tools and norms.

Secondly, theories of change underpinning interventions and defining what the endpoint of any investment is hoped to be, will be an important area of work. Articulating assumptions about what constitutes resilience or recovery in a particular location will be inherently political and value-based, including defining what this means for different groups, over what timeframes and conditions. Wellbeing outcomes will vary depending on the type of intervention, for example, those focused on commemoration and/or recognition of loss may be focused on repair to individual and collective identities. The measurement processes in adaptation MERL have at times left the pathways to change under-explored, creating a black box between aspiration and longer-term impact^53^, as well as offering limited data on the sustainability of responses beyond project closure^66,83^. Loss and damage practitioners will need to continually challenge their assumptions and be clear about the role of knowledge production beyond projectized MERL systems to generate new ways of understanding changes and effective responses.

Lastly, the initial conceptualisation of adaptation MERL and more recent work on the locally led adaptation principles has emphasised the importance of the process of data collection and wider participation of local communities^68^. However, the pressures on many MERL systems has often resulted in more traditional forms of extractive data collection to fulfil reporting requirements^84,85^. The emerging work on the science of loss emphasises the importance of the process as being potentially transformative, and with some types of loss and damage projects it could be the process of recording loss is as much about recognising and processing trauma and commemoration as the data that is collected. This therefore puts the impetus on loss and damage scholars and practitioners to develop the normative principles that should underpin knowledge processes around loss and damage to ensure that knowledge production is not re-traumatising those affected but offers opportunities for agency and accountability.

In conclusion, we caution loss and damage scholars and practitioners from allowing the dominant narratives on results-based management and audit culture to frame how a MERL system might support projects responding to loss and damage. We argue that scholars and practitioners need to be deliberate in choosing the appropriate scale for any MERL effort linked to its primary purpose and be aware of the trade-offs for the policy and scholarly community between focusing on global, national and local evidence needs. Debates on adaptation MERL began over a decade ago including debates on outcomes and effectiveness^1,6^. However, despite years of experience there is limited data available on longer-term outcomes and many systems have been designed to report upwards rather than performing a more transformative role for those affected. Loss and damage projects are inherently part of a justice-based agenda, and as such the process of tracking and learning from projects should seek to align with these ideals. There is a now a short window of opportunity to shape MERL in service of those experiencing loss and damage rather than only those funding the responses.

## Data Availability

No datasets were generated or analysed during the current study.
